# Pr_16_Mo_21_O_56_

**DOI:** 10.1107/S1600536811015649

**Published:** 2011-04-29

**Authors:** Patrick Gougeon, Philippe Gall

**Affiliations:** a Sciences Chimiques de Rennes, UMR CNRS No. 6226, Université de Rennes I – INSA Rennes, Avenue du Général Leclerc, 35042 Rennes CEDEX, France

## Abstract

The structure of hexa­deca­praseodymium henicosa­molybden­um hexa­penta­contaoxide, Pr_16_Mo_21_O_56_, is isotypic with other rare earth representatives of formula type *RE*_16_Mo_21_O_56_ (*RE* = La, Ce, Nd). It is characterized by Mo_10_O_18_^*i*^O_8_^*a*^ units (where *i* = inner and *a* = apical O atoms) containing biocta­hedral Mo_10_ clusters and octa­hedral MoO_6_ units that share some of their O atoms to form the Mo–O framework. The two independent Mo_10_ cluster units are centred at Wyckoff positions 2*b* and 2*c* and have point-group symmetry 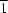. The Mo atom of the MoO_6_ unit is likewise situated at an inversion centre (2*d*). The eight crystallographically different Pr^3+^ cations occupy irregular voids in the framework with coordination numbers to the O atoms varying between 8 and 11.

## Related literature

For previous reports on the crystal structures of *RE*_16_Mo_21_O_56_ compounds, see: Gall & Gougeon (1993 ▶) for *RE* = Ce; Gall & Gougeon (1998 ▶) for *RE* = Nd; Gall *et al.* (1999 ▶) for *RE* = La. For details of the *i*- and *a*-type ligand notation, see: Schäfer & von Schnering (1964 ▶).

## Experimental

### 

#### Crystal data

Pr_16_Mo_21_O_56_
*M* *_r_* = 5165.30
Monoclinic, 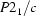

*a* = 13.427 (3) Å
*b* = 13.3935 (16) Å
*c* = 13.318 (3) Å
β = 100.102 (13)°
*V* = 2357.9 (8) Å^3^
*Z* = 2
Mo *K*α radiation
μ = 21.65 mm^−1^
*T* = 293 K
0.10 × 0.07 × 0.03 mm

#### Data collection

Nonius KappaCCD diffractometer
Absorption correction: multi-scan (*PLATON*; Spek, 2009 ▶) *T*_min_ = 0.060, *T*_max_ = 0.216
97514 measured reflections
18339 independent reflections
15545 reflections with *I* > 2σ(*I*)
*R*_int_ = 0.040

#### Refinement

*R*[*F* ^2^ > 2σ(*F* ^2^)] = 0.025
*wR*(*F* ^2^) = 0.049
*S* = 1.13
18339 reflections
422 parameters
Δρ_max_ = 2.19 e Å^−3^
Δρ_min_ = −2.34 e Å^−3^

### 

Data collection: *COLLECT* (Nonius, 1998 ▶); cell refinement: *COLLECT*; data reduction: *EVALCCD* (Duisenberg *et al.*, 2003 ▶); program(s) used to solve structure: *SIR97* (Altomare *et al.*, 1999 ▶); program(s) used to refine structure: *SHELXL97* (Sheldrick, 2008 ▶); molecular graphics: *DIAMOND* (Bergerhoff, 1996 ▶); software used to prepare material for publication: *SHELXL97*.

## Supplementary Material

Crystal structure: contains datablocks I, global. DOI: 10.1107/S1600536811015649/wm2479sup1.cif

Structure factors: contains datablocks I. DOI: 10.1107/S1600536811015649/wm2479Isup2.hkl

Additional supplementary materials:  crystallographic information; 3D view; checkCIF report

## Figures and Tables

**Table 1:** Selected bond lengths (Å)

Pr1—O28^i^	2.320 (2)
Pr1—O22	2.358 (2)
Pr1—O3	2.489 (2)
Pr1—O2	2.497 (2)
Pr1—O6^ii^	2.599 (2)
Pr1—O10	2.628 (2)
Pr1—O4^ii^	2.695 (2)
Pr1—O9^ii^	2.897 (2)
Pr1—O17	3.012 (2)
Pr1—O19	3.212 (2)
Pr2—O13^ii^	2.313 (2)
Pr2—O26	2.382 (2)
Pr2—O28^i^	2.392 (2)
Pr2—O16^iii^	2.461 (2)
Pr2—O15^iii^	2.513 (2)
Pr2—O23^iii^	2.6186 (19)
Pr2—O14	2.675 (2)
Pr2—O19	2.739 (2)
Pr2—O1^ii^	2.914 (2)
Pr3—O5^ii^	2.378 (2)
Pr3—O28^i^	2.396 (2)
Pr3—O6^iv^	2.428 (2)
Pr3—O19^i^	2.497 (2)
Pr3—O21^i^	2.499 (2)
Pr3—O10	2.835 (2)
Pr3—O1^ii^	2.836 (2)
Pr3—O8^iv^	2.854 (2)
Pr3—O22^i^	3.166 (2)
Pr3—O23^iii^	3.225 (2)
Pr3—O4^ii^	3.253 (2)
Pr4—O18	2.360 (2)
Pr4—O26^i^	2.3740 (19)
Pr4—O13^iv^	2.399 (2)
Pr4—O12	2.437 (2)
Pr4—O28^i^	2.563 (2)
Pr4—O25^iii^	2.723 (2)
Pr4—O18^v^	2.854 (2)
Pr4—O23^iii^	3.051 (2)
Pr4—O14	3.235 (2)
Pr4—O17	3.411 (2)
Pr4—O10	3.439 (2)
Pr5—O8^ii^	2.293 (2)
Pr5—O27^vi^	2.293 (2)
Pr5—O20^vii^	2.364 (2)
Pr5—O2^vii^	2.372 (2)
Pr5—O22^vii^	2.572 (2)
Pr5—O5^vii^	2.629 (2)
Pr5—O9^ii^	2.841 (2)
Pr5—O4^vii^	3.005 (2)
Pr5—O17^vii^	3.128 (2)
Pr6—O25^ii^	2.226 (2)
Pr6—O11^iv^	2.367 (2)
Pr6—O16^viii^	2.3794 (19)
Pr6—O12^iv^	2.485 (2)
Pr6—O18^viii^	2.602 (2)
Pr6—O14^viii^	2.752 (2)
Pr6—O27^ix^	2.753 (2)
Pr6—O13	2.797 (2)
Pr7—O12	2.331 (2)
Pr7—O26^x^	2.352 (2)
Pr7—O15	2.442 (2)
Pr7—O25^xi^	2.465 (2)
Pr7—O27^i^	2.474 (2)
Pr7—O24^xi^	2.598 (2)
Pr7—O18	2.675 (2)
Pr7—O17	2.810 (2)
Pr7—O7	3.128 (2)
Pr8—O27^ix^	2.309 (2)
Pr8—O21^vii^	2.384 (2)
Pr8—O9^ii^	2.389 (2)
Pr8—O3	2.442 (2)
Pr8—O7^ii^	2.509 (2)
Pr8—O20^vii^	2.700 (2)
Pr8—O24^vii^	2.708 (2)
Pr8—O4^ii^	3.235 (2)
Pr8—O1	3.360 (2)
Mo1—O1	1.939 (2)
Mo1—O4	2.004 (2)
Mo1—O3	2.009 (2)
Mo1—O2	2.046 (2)
Mo1—O5	2.175 (2)
Mo1—Mo2	2.6435 (5)
Mo1—Mo4	2.7048 (6)
Mo1—Mo5	2.7050 (4)
Mo1—Mo3	2.8049 (7)
Mo2—O7	1.973 (2)
Mo2—O8	1.979 (2)
Mo2—O4	2.008 (2)
Mo2—O6	2.019 (2)
Mo2—O9	2.159 (2)
Mo2—Mo5^iv^	2.7076 (5)
Mo2—Mo3	2.7191 (4)
Mo2—Mo4	2.7525 (5)
Mo3—O12	2.032 (2)
Mo3—O2	2.0508 (19)
Mo3—O7	2.077 (2)
Mo3—O10	2.095 (2)
Mo3—O11	2.0981 (19)
Mo3—Mo4^iv^	2.6477 (4)
Mo3—Mo5	2.7423 (5)
Mo3—Mo5^iv^	2.7819 (6)
Mo4—O13	1.963 (2)
Mo4—O1	2.083 (2)
Mo4—O11^iv^	2.088 (2)
Mo4—O6	2.088 (2)
Mo4—O10^iv^	2.0982 (19)
Mo4—Mo3^iv^	2.6477 (4)
Mo4—Mo5^iv^	2.7530 (6)
Mo4—Mo5	2.7756 (4)
Mo5—O8^iv^	2.018 (2)
Mo5—O11^iv^	2.045 (2)
Mo5—O3	2.0506 (19)
Mo5—O10	2.061 (2)
Mo5—Mo2^iv^	2.7076 (5)
Mo5—Mo4^iv^	2.7530 (6)
Mo5—Mo3^iv^	2.7819 (6)
Mo5—Mo5^iv^	2.8260 (6)
Mo6—O14	1.955 (2)
Mo6—O17	1.987 (2)
Mo6—O15	2.016 (2)
Mo6—O16	2.025 (2)
Mo6—O18	2.143 (2)
Mo6—Mo7	2.6087 (6)
Mo6—Mo9	2.7077 (4)
Mo6—Mo10^xii^	2.7396 (6)
Mo6—Mo8	2.7987 (5)
Mo7—O17	1.980 (2)
Mo7—O20	1.991 (2)
Mo7—O19	1.991 (2)
Mo7—O21	2.0146 (19)
Mo7—O22	2.133 (2)
Mo7—Mo10	2.7151 (6)
Mo7—Mo9	2.7257 (5)
Mo7—Mo8	2.7514 (4)
Mo8—O25	2.025 (2)
Mo8—O15	2.0509 (19)
Mo8—O20	2.075 (2)
Mo8—O24	2.0843 (19)
Mo8—O23	2.088 (2)
Mo8—Mo9^xii^	2.6006 (6)
Mo8—Mo10^xii^	2.7556 (5)
Mo8—Mo10	2.7648 (4)
Mo9—O26	1.955 (2)
Mo9—O19	2.064 (2)
Mo9—O14	2.084 (2)
Mo9—O23^xii^	2.0935 (19)
Mo9—O24^xii^	2.105 (2)
Mo9—Mo8^xii^	2.6006 (6)
Mo9—Mo10	2.7168 (5)
Mo9—Mo10^xii^	2.7422 (4)
Mo10—O16^xii^	2.0182 (19)
Mo10—O21	2.062 (2)
Mo10—O23^xii^	2.066 (2)
Mo10—O24	2.086 (2)
Mo10—Mo6^xii^	2.7396 (6)
Mo10—Mo9^xii^	2.7422 (4)
Mo10—Mo8^xii^	2.7556 (5)
Mo10—Mo10^xii^	2.8525 (7)
Mo11—O5^vii^	2.024 (2)
Mo11—O5	2.024 (2)
Mo11—O9^xiii^	2.027 (2)
Mo11—O9^ii^	2.027 (2)
Mo11—O22	2.048 (2)
Mo11—O22^vii^	2.048 (2)

Symmetry codes: (i) 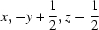; (ii) 
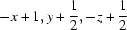
; (iii) 
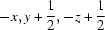
; (iv) 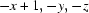; (v) 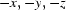; (vi) 
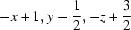
; (vii) 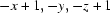; (viii) 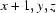; (ix) 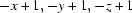; (x) 
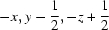
; (xi) 
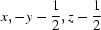
; (xii) 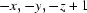; (xiii) 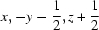.

**Fig. 1.**
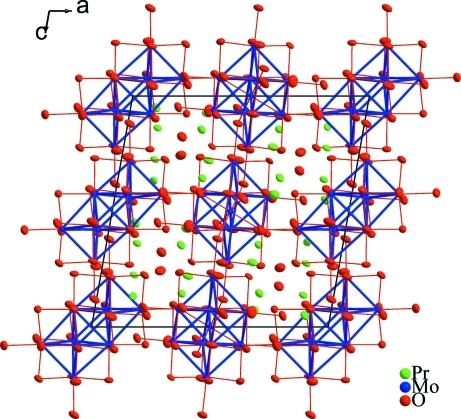
: Projection of the crystal structure of Pr16Mo21O56 on the ac plane. Displacement ellipsoids are drawn at the 97% probability level.

**Fig. 2.**
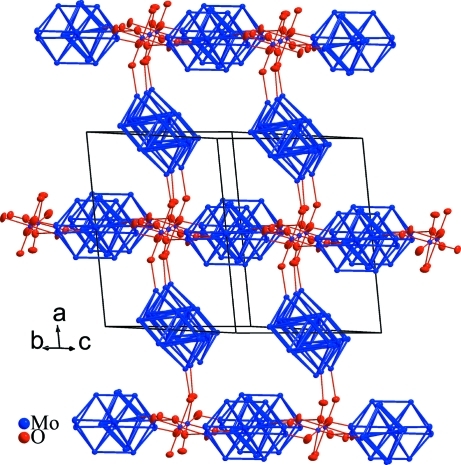
: The interunit linkage through the apical oxygen atoms between the Mo10 clusters and single Mo atoms.

**Fig. 3.**
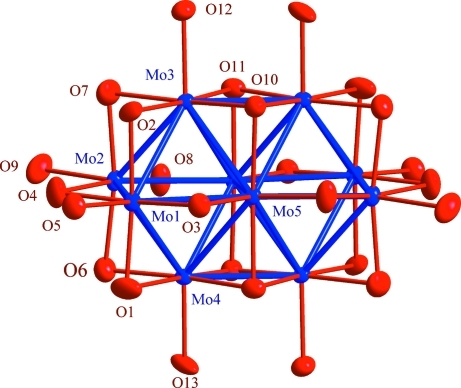
: Plot showing the atom-numbering scheme of the Mo10O18iO8<a/i>a unit (Mo10(I)). Displacement ellipsoids are drawn at the 97% probability level.

**Fig. 4.**
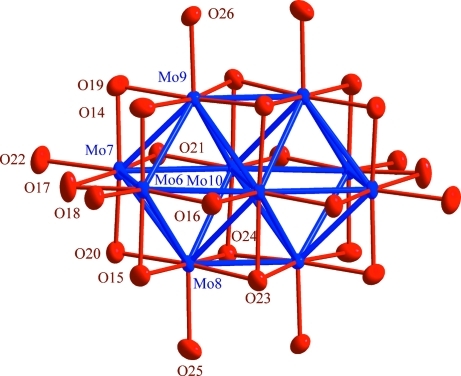
: Plot showing the atom-numbering scheme of the Mo10O18iO8<a/i>a unit (Mo10(II)). Displacement ellipsoids are drawn at the 97% probability level.

## supplementary crystallographic information

### Comment

Pr_16_Mo_21_O_56_ is isotypic with the Ce_16_Mo_21_O_56_ (Gall & Gougeon, 1993) structure type that has been described in the early 1990s. Subsequently, we also reported the crystal structures of the Nd (Gall & Gougeon, 1998) and La (Gall *et al.*, 1999) members of this formula type. The crystal structure is based on Mo_10_O_18_*^i^*O_8_*^a^* cluster units sharing two or four apical O*^a^* atoms with adjacent MoO_6_ octahedra (Figs. 1 and 2). For details of the *i*- and *a*-type ligand notation, see: Schäfer & von Schnering (1964). The Mo core of the two crystallographically independent Mo_10_O_18_*^i^*O_8_*^a^* units results from metal edge-sharing of two Mo_6_ octahedra. The Mo_10_(I) cluster formed by the Mo1, Mo2, Mo3, Mo4 and Mo5 atoms are centred on 2*b* position and the Mo_10_(II) cluster formed by the Mo6, Mo7, Mo8, Mo9 and Mo10 atoms on 2*c* position, while the single Mo11 atoms occupy the inversion centres located at the center of four of the six faces (2*d* position) of the unit-cell. Figure 2 shows the interunit linkage through the apical oxygen atoms between the Mo_10_(II) clusters and single Mo atoms within the slabs parallel to the *bc* plane. One can notice that the Mo_10_(II) clusters are linked to four single Mo atoms, whereas the Mo10(I) ones are only linked to two Mo atoms through oxygen atoms. Consequently, the connectivity formula for the Mo_10_O_18_*^i^*O_8_*^a^* and MoO_6_ units can be described as follows: MoO_6/2_Mo_10_O_18_*^i^*O_4_*^a^*O_4/2_*^a-a^*- Mo_10_O_18_*^i^*O_6_*^a^*O_2/2_*^a-a^*. In the Mo_10_O_18_*^i^*O_8_*^a^* cluster units present in Pr_16_Mo_21_O_56_, the Mo—Mo distances range between 2.6435 (5) and 2.8260 (6) Å and between 2.6087 (6) and 2.8525 (7) Å in the Mo_10_ clusters I and II, respectively. With the exception of Mo5 and Mo10, which are shared by both octahedra forming the Mo10 clusters and are surrounded by four O atoms, all the other Mo atoms are bonded to five O atoms in an approximately square-pyramidal environment (Figs. 3, 4). The Mo—O distances lie between 1.955 (2) and 2.143 (2) Å in cluster I and between 1.939 (2) and 2.175 (2) Å in cluster II. The single Mo atoms are surrounded by six O atoms forming a slightly tetragonally distorted octahedron with four oxygen atoms [2× O5 and 2× O9] at 2.024 (2) and 2.027 (2) Å and two O22 atoms at 2.048 (2) Å. All the eight different Pr^3+^ ions are in general positions and occupy irregularly O-coordinated sites inbetween the Mo_10_O_18_*^i^*O_8_*^a^* units. The coordination numbers of the Pr^3+^ ions vary from 8 to 11 with Pr—O distances spreading over a wide range [2.226 (2) to 3.439 (2) Å].

### Experimental

Single crystals of Pr_16_Mo_21_O_56_ were obtained from a mixture of Pr_6_O_11_, MoO_3_, and Mo with a nominal composition Pr_2_Mo_2_O_7_. Before use, Mo powder was reduced under H_2_ flowing gas at 1273 K during ten hours in order to eliminate any trace of oxygen. The initial mixture (ca 4 g) was cold pressed and loaded into a molybdenum crucible, which was sealed under a low argon pressure using an arc welding system. The charge was heated at the rate of 300 K/h up to 2000 K, the temperature which was held for 18 hours, then cooled at 100 K/h down to 1373 K and finally furnace cooled.

### Refinement

The highest peak and the deepest hole are located 0.636 Å and 0.70 Å from Pr1 and Pr2, respectively.

### Crystal data

**Table:**

Pr_16_Mo_21_O_56_	*F*(000) = 4548
*M**_r_* = 5165.30	*D*_x_ = 7.275 Mg m^−^^3^
Monoclinic, *P*2_1_/*c*	Mo *K*α radiation, λ = 0.71073 Å
Hall symbol: -P 2ybc	Cell parameters from 96298 reflections
*a* = 13.427 (3) Å	θ = 3.5–44.0°
*b* = 13.3935 (16) Å	µ = 21.65 mm^−^^1^
*c* = 13.318 (3) Å	*T* = 293 K
β = 100.102 (13)°	Irregular block, black
*V* = 2357.9 (8) Å^3^	0.10 × 0.07 × 0.03 mm
*Z* = 2	

### Data collection

**Table:**

Nonius KappaCCD diffractometer	18339 independent reflections
Radiation source: fine-focus sealed tube	15545 reflections with *I* > 2σ(*I*)
graphite	*R*_int_ = 0.040
φ scans (κ = 0) + additional ω scans	θ_max_ = 44.0°, θ_min_ = 3.5°
Absorption correction: multi-scan (*PLATON*; Spek, 2009)	*h* = −21→26
*T*_min_ = 0.060, *T*_max_ = 0.216	*k* = −26→24
97514 measured reflections	*l* = −26→19

### Refinement

**Table:**

Refinement on *F*^2^	Primary atom site location: structure-invariant direct methods
Least-squares matrix: full	Secondary atom site location: difference Fourier map
*R*[*F*^2^ > 2σ(*F*^2^)] = 0.025	*w* = 1/[σ^2^(*F*_o_^2^) + (0.0113*P*)^2^ + 11.0419*P*] where *P* = (*F*_o_^2^ + 2*F*_c_^2^)/3
*wR*(*F*^2^) = 0.049	(Δ/σ)_max_ = 0.003
*S* = 1.13	Δρ_max_ = 2.19 e Å^−^^3^
18339 reflections	Δρ_min_ = −2.34 e Å^−^^3^
422 parameters	Extinction correction: *SHELXL97* (Sheldrick, 2008), Fc^*^=kFc[1+0.001xFc^2^λ^3^/sin(2θ)]^-1/4^
0 restraints	Extinction coefficient: 0.001364 (15)

### Special details

**Table:**

Geometry. All esds (except the esd in the dihedral angle between two l.s. planes) are estimated using the full covariance matrix. The cell esds are taken into account individually in the estimation of esds in distances, angles and torsion angles; correlations between esds in cell parameters are only used when they are defined by crystal symmetry. An approximate (isotropic) treatment of cell esds is used for estimating esds involving l.s. planes.
Refinement. Refinement of F^2^ against ALL reflections. The weighted R-factor wR and goodness of fit S are based on F^2^, conventional R-factors R are based on F, with F set to zero for negative F^2^. The threshold expression of F^2^ > 2sigma(F^2^) is used only for calculating R-factors(gt) etc. and is not relevant to the choice of reflections for refinement. R-factors based on F^2^ are statistically about twice as large as those based on F, and R- factors based on ALL data will be even larger.

### Fractional atomic coordinates and isotropic or equivalent isotropic displacement parameters (Å^2^)

**Table:**

	*x*	*y*	*z*	*U*_iso_*/*U*_eq_	
Pr1	0.368587 (12)	0.120261 (11)	0.278756 (11)	0.00606 (2)	
Pr2	0.141006 (11)	0.302761 (11)	0.274727 (10)	0.00475 (2)	
Pr3	0.326661 (12)	0.309727 (11)	0.073640 (11)	0.00650 (2)	
Pr4	0.107285 (12)	0.110561 (11)	0.049623 (11)	0.00605 (2)	
Pr5	0.644480 (11)	0.164018 (11)	0.644218 (10)	0.00498 (2)	
Pr6	0.847572 (11)	0.096160 (12)	0.133086 (11)	0.00657 (2)	
Pr7	0.129504 (11)	−0.167711 (11)	0.137964 (10)	0.00478 (2)	
Pr8	0.683495 (11)	0.126717 (11)	0.354490 (11)	0.00589 (2)	
Mo1	0.523977 (17)	−0.076331 (16)	0.216424 (15)	0.00341 (3)	
Mo2	0.482272 (17)	−0.210959 (16)	0.069929 (16)	0.00369 (3)	
Mo3	0.353802 (16)	−0.053160 (16)	0.062708 (15)	0.00313 (3)	
Mo4	0.647517 (16)	−0.087908 (16)	0.076793 (15)	0.00332 (3)	
Mo5	0.516970 (16)	0.072571 (16)	0.078141 (15)	0.00293 (3)	
Mo6	0.038623 (17)	−0.005493 (16)	0.282727 (15)	0.00332 (3)	
Mo7	0.198702 (16)	0.008403 (16)	0.424741 (16)	0.00342 (3)	
Mo8	0.067857 (16)	−0.146721 (16)	0.440040 (15)	0.00309 (3)	
Mo9	0.046379 (17)	0.143583 (16)	0.421214 (15)	0.00306 (3)	
Mo10	0.087885 (16)	0.010377 (16)	0.576784 (15)	0.00292 (3)	
Mo11	0.5000	0.0000	0.5000	0.00293 (4)	
O1	0.66926 (16)	−0.09527 (17)	0.23536 (15)	0.0077 (3)	
O2	0.37338 (15)	−0.05606 (16)	0.21890 (14)	0.0057 (3)	
O3	0.53464 (15)	0.07242 (15)	0.23423 (14)	0.0051 (3)	
O4	0.50407 (17)	−0.22434 (15)	0.22242 (15)	0.0071 (3)	
O5	0.53471 (16)	−0.07903 (15)	0.38128 (14)	0.0060 (3)	
O6	0.63101 (16)	−0.24270 (15)	0.08297 (15)	0.0063 (3)	
O7	0.33446 (17)	−0.20703 (15)	0.06372 (16)	0.0077 (3)	
O8	0.47829 (17)	−0.22315 (15)	−0.07884 (14)	0.0067 (3)	
O9	0.45758 (17)	−0.37016 (15)	0.05837 (16)	0.0078 (3)	
O10	0.36696 (15)	0.10183 (15)	0.08203 (14)	0.0051 (3)	
O11	0.32871 (15)	−0.06541 (15)	−0.09680 (14)	0.0051 (3)	
O12	0.20085 (15)	−0.04524 (15)	0.04837 (15)	0.0061 (3)	
O13	0.79415 (16)	−0.10203 (17)	0.08399 (15)	0.0086 (3)	
O14	0.02575 (16)	0.13881 (15)	0.26249 (15)	0.0064 (3)	
O15	0.04258 (16)	−0.15598 (15)	0.28392 (14)	0.0054 (3)	
O16	−0.11323 (15)	−0.01986 (15)	0.26960 (14)	0.0051 (3)	
O17	0.18509 (16)	−0.00990 (16)	0.27544 (15)	0.0072 (3)	
O18	0.00836 (16)	−0.00776 (15)	0.11934 (15)	0.0066 (3)	
O19	0.19877 (15)	0.15686 (15)	0.41721 (15)	0.0060 (3)	
O20	0.22338 (16)	−0.13688 (15)	0.44890 (15)	0.0060 (3)	
O21	0.24076 (15)	0.02508 (15)	0.57660 (14)	0.0050 (3)	
O22	0.35634 (16)	0.01724 (16)	0.41968 (15)	0.0068 (3)	
O23	−0.08880 (15)	−0.16463 (15)	0.42132 (14)	0.0052 (3)	
O24	0.11258 (15)	−0.14253 (15)	0.59797 (14)	0.0048 (3)	
O25	0.07419 (17)	−0.29717 (15)	0.45501 (15)	0.0071 (3)	
O26	0.04233 (15)	0.28883 (15)	0.40629 (14)	0.0051 (3)	
O27	0.24825 (17)	0.77237 (16)	0.75659 (15)	0.0077 (3)	
O28	0.23897 (16)	0.29374 (16)	0.67630 (15)	0.0072 (3)	

### Atomic displacement parameters (Å^2^)

**Table:**

	*U*^11^	*U*^22^	*U*^33^	*U*^12^	*U*^13^	*U*^23^
Pr1	0.00832 (6)	0.00590 (5)	0.00427 (4)	0.00187 (4)	0.00198 (4)	0.00071 (4)
Pr2	0.00594 (5)	0.00528 (5)	0.00344 (4)	0.00063 (4)	0.00193 (4)	−0.00011 (4)
Pr3	0.00778 (5)	0.00660 (5)	0.00530 (5)	−0.00091 (4)	0.00161 (4)	0.00035 (4)
Pr4	0.00620 (5)	0.00635 (5)	0.00614 (5)	0.00004 (4)	0.00258 (4)	0.00140 (4)
Pr5	0.00504 (5)	0.00558 (5)	0.00447 (4)	−0.00028 (4)	0.00125 (4)	−0.00010 (4)
Pr6	0.00451 (5)	0.01048 (6)	0.00472 (5)	−0.00097 (4)	0.00085 (4)	0.00316 (4)
Pr7	0.00554 (5)	0.00556 (5)	0.00349 (4)	−0.00040 (4)	0.00146 (4)	0.00016 (4)
Pr8	0.00541 (5)	0.00732 (5)	0.00531 (5)	0.00055 (4)	0.00198 (4)	0.00138 (4)
Mo1	0.00429 (7)	0.00431 (7)	0.00188 (6)	0.00019 (6)	0.00121 (5)	−0.00003 (6)
Mo2	0.00500 (8)	0.00326 (7)	0.00312 (7)	−0.00003 (6)	0.00159 (6)	−0.00004 (6)
Mo3	0.00299 (7)	0.00406 (7)	0.00254 (6)	−0.00017 (6)	0.00101 (5)	0.00002 (6)
Mo4	0.00302 (7)	0.00462 (7)	0.00251 (6)	0.00034 (6)	0.00098 (5)	0.00004 (6)
Mo5	0.00336 (7)	0.00351 (7)	0.00215 (6)	−0.00021 (6)	0.00109 (5)	−0.00029 (5)
Mo6	0.00389 (7)	0.00417 (7)	0.00209 (6)	0.00009 (6)	0.00109 (5)	−0.00002 (5)
Mo7	0.00290 (7)	0.00462 (7)	0.00300 (7)	0.00007 (6)	0.00123 (5)	0.00014 (6)
Mo8	0.00370 (7)	0.00354 (7)	0.00227 (6)	0.00027 (6)	0.00121 (5)	0.00008 (5)
Mo9	0.00366 (7)	0.00310 (7)	0.00260 (6)	−0.00003 (6)	0.00107 (5)	0.00022 (5)
Mo10	0.00317 (7)	0.00366 (7)	0.00212 (6)	0.00003 (6)	0.00098 (5)	0.00005 (5)
Mo11	0.00315 (10)	0.00371 (10)	0.00208 (9)	−0.00019 (8)	0.00090 (8)	−0.00004 (8)
O1	0.0054 (7)	0.0140 (9)	0.0035 (6)	0.0009 (6)	0.0002 (5)	−0.0003 (6)
O2	0.0048 (7)	0.0096 (8)	0.0032 (6)	−0.0007 (6)	0.0022 (5)	0.0009 (6)
O3	0.0067 (7)	0.0055 (7)	0.0033 (6)	0.0001 (6)	0.0011 (5)	−0.0011 (5)
O4	0.0109 (8)	0.0066 (7)	0.0040 (6)	0.0001 (6)	0.0020 (6)	0.0010 (6)
O5	0.0077 (8)	0.0071 (7)	0.0036 (6)	0.0008 (6)	0.0021 (5)	−0.0004 (5)
O6	0.0073 (8)	0.0058 (7)	0.0065 (7)	0.0014 (6)	0.0030 (6)	0.0005 (6)
O7	0.0077 (8)	0.0043 (7)	0.0112 (8)	−0.0009 (6)	0.0017 (6)	−0.0003 (6)
O8	0.0120 (8)	0.0049 (7)	0.0034 (6)	−0.0009 (6)	0.0018 (6)	−0.0008 (5)
O9	0.0104 (8)	0.0050 (7)	0.0093 (7)	−0.0005 (6)	0.0060 (6)	−0.0021 (6)
O10	0.0054 (7)	0.0057 (7)	0.0045 (6)	0.0002 (6)	0.0016 (5)	0.0000 (5)
O11	0.0048 (7)	0.0068 (7)	0.0037 (6)	0.0004 (6)	0.0010 (5)	−0.0010 (5)
O12	0.0049 (7)	0.0070 (7)	0.0070 (7)	0.0002 (6)	0.0027 (5)	0.0002 (6)
O13	0.0052 (7)	0.0150 (9)	0.0063 (7)	0.0051 (7)	0.0028 (6)	0.0041 (6)
O14	0.0095 (8)	0.0048 (7)	0.0046 (6)	0.0001 (6)	0.0009 (6)	0.0008 (5)
O15	0.0078 (7)	0.0058 (7)	0.0028 (6)	−0.0004 (6)	0.0016 (5)	−0.0002 (5)
O16	0.0062 (7)	0.0064 (7)	0.0027 (6)	−0.0006 (6)	0.0006 (5)	−0.0002 (5)
O17	0.0060 (7)	0.0117 (8)	0.0043 (6)	−0.0007 (6)	0.0025 (5)	−0.0007 (6)
O18	0.0086 (8)	0.0073 (7)	0.0048 (6)	−0.0005 (6)	0.0032 (6)	0.0002 (6)
O19	0.0051 (7)	0.0054 (7)	0.0075 (7)	−0.0008 (6)	0.0011 (6)	0.0009 (6)
O20	0.0055 (7)	0.0059 (7)	0.0072 (7)	0.0008 (6)	0.0027 (6)	0.0013 (6)
O21	0.0041 (7)	0.0067 (7)	0.0042 (6)	−0.0007 (6)	0.0008 (5)	−0.0006 (5)
O22	0.0045 (7)	0.0096 (8)	0.0065 (7)	−0.0005 (6)	0.0015 (5)	0.0013 (6)
O23	0.0061 (7)	0.0056 (7)	0.0041 (6)	−0.0001 (6)	0.0015 (5)	−0.0010 (5)
O24	0.0053 (7)	0.0050 (7)	0.0041 (6)	0.0003 (5)	0.0011 (5)	0.0006 (5)
O25	0.0111 (8)	0.0053 (7)	0.0056 (7)	0.0012 (6)	0.0031 (6)	−0.0003 (6)
O26	0.0066 (7)	0.0049 (7)	0.0042 (6)	−0.0011 (6)	0.0017 (5)	0.0007 (5)
O27	0.0082 (8)	0.0087 (8)	0.0060 (7)	−0.0007 (6)	0.0004 (6)	0.0017 (6)
O28	0.0072 (8)	0.0080 (8)	0.0066 (7)	0.0000 (6)	0.0022 (6)	−0.0008 (6)

### Geometric parameters (Å, °)

**Table:**

Pr1—O28^i^	2.320 (2)	Mo1—Mo4	2.7048 (6)
Pr1—O22	2.358 (2)	Mo1—Mo5	2.7050 (4)
Pr1—O3	2.489 (2)	Mo1—Mo3	2.8049 (7)
Pr1—O2	2.497 (2)	Mo2—O7	1.973 (2)
Pr1—O6^ii^	2.599 (2)	Mo2—O8	1.979 (2)
Pr1—O10	2.628 (2)	Mo2—O4	2.008 (2)
Pr1—O4^ii^	2.695 (2)	Mo2—O6	2.019 (2)
Pr1—O9^ii^	2.897 (2)	Mo2—O9	2.159 (2)
Pr1—O17	3.012 (2)	Mo2—Mo5^iv^	2.7076 (5)
Pr1—O19	3.212 (2)	Mo2—Mo3	2.7191 (4)
Pr2—O13^ii^	2.313 (2)	Mo2—Mo4	2.7525 (5)
Pr2—O26	2.382 (2)	Mo3—O12	2.032 (2)
Pr2—O28^i^	2.392 (2)	Mo3—O2	2.0508 (19)
Pr2—O16^iii^	2.461 (2)	Mo3—O7	2.077 (2)
Pr2—O15^iii^	2.513 (2)	Mo3—O10	2.095 (2)
Pr2—O23^iii^	2.6186 (19)	Mo3—O11	2.0981 (19)
Pr2—O14	2.675 (2)	Mo3—Mo4^iv^	2.6477 (4)
Pr2—O19	2.739 (2)	Mo3—Mo5	2.7423 (5)
Pr2—O1^ii^	2.914 (2)	Mo3—Mo5^iv^	2.7819 (6)
Pr3—O5^ii^	2.378 (2)	Mo4—O13	1.963 (2)
Pr3—O28^i^	2.396 (2)	Mo4—O1	2.083 (2)
Pr3—O6^iv^	2.428 (2)	Mo4—O11^iv^	2.088 (2)
Pr3—O19^i^	2.497 (2)	Mo4—O6	2.088 (2)
Pr3—O21^i^	2.499 (2)	Mo4—O10^iv^	2.0982 (19)
Pr3—O10	2.835 (2)	Mo4—Mo3^iv^	2.6477 (4)
Pr3—O1^ii^	2.836 (2)	Mo4—Mo5^iv^	2.7530 (6)
Pr3—O8^iv^	2.854 (2)	Mo4—Mo5	2.7756 (4)
Pr3—O22^i^	3.166 (2)	Mo5—O8^iv^	2.018 (2)
Pr3—O23^iii^	3.225 (2)	Mo5—O11^iv^	2.045 (2)
Pr3—O4^ii^	3.253 (2)	Mo5—O3	2.0506 (19)
Pr4—O18	2.360 (2)	Mo5—O10	2.061 (2)
Pr4—O26^i^	2.3740 (19)	Mo5—Mo2^iv^	2.7076 (5)
Pr4—O13^iv^	2.399 (2)	Mo5—Mo4^iv^	2.7530 (6)
Pr4—O12	2.437 (2)	Mo5—Mo3^iv^	2.7819 (6)
Pr4—O28^i^	2.563 (2)	Mo5—Mo5^iv^	2.8260 (6)
Pr4—O25^iii^	2.723 (2)	Mo6—O14	1.955 (2)
Pr4—O18^v^	2.854 (2)	Mo6—O17	1.987 (2)
Pr4—O23^iii^	3.051 (2)	Mo6—O15	2.016 (2)
Pr4—O14	3.235 (2)	Mo6—O16	2.025 (2)
Pr4—O17	3.411 (2)	Mo6—O18	2.143 (2)
Pr4—O10	3.439 (2)	Mo6—Mo7	2.6087 (6)
Pr5—O8^ii^	2.293 (2)	Mo6—Mo9	2.7077 (4)
Pr5—O27^vi^	2.293 (2)	Mo6—Mo10^xii^	2.7396 (6)
Pr5—O20^vii^	2.364 (2)	Mo6—Mo8	2.7987 (5)
Pr5—O2^vii^	2.372 (2)	Mo7—O17	1.980 (2)
Pr5—O22^vii^	2.572 (2)	Mo7—O20	1.991 (2)
Pr5—O5^vii^	2.629 (2)	Mo7—O19	1.991 (2)
Pr5—O9^ii^	2.841 (2)	Mo7—O21	2.0146 (19)
Pr5—O4^vii^	3.005 (2)	Mo7—O22	2.133 (2)
Pr5—O17^vii^	3.128 (2)	Mo7—Mo10	2.7151 (6)
Pr6—O25^ii^	2.226 (2)	Mo7—Mo9	2.7257 (5)
Pr6—O11^iv^	2.367 (2)	Mo7—Mo8	2.7514 (4)
Pr6—O16^viii^	2.3794 (19)	Mo8—O25	2.025 (2)
Pr6—O12^iv^	2.485 (2)	Mo8—O15	2.0509 (19)
Pr6—O18^viii^	2.602 (2)	Mo8—O20	2.075 (2)
Pr6—O14^viii^	2.752 (2)	Mo8—O24	2.0843 (19)
Pr6—O27^ix^	2.753 (2)	Mo8—O23	2.088 (2)
Pr6—O13	2.797 (2)	Mo8—Mo9^xii^	2.6006 (6)
Pr7—O12	2.331 (2)	Mo8—Mo10^xii^	2.7556 (5)
Pr7—O26^x^	2.352 (2)	Mo8—Mo10	2.7648 (4)
Pr7—O15	2.442 (2)	Mo9—O26	1.955 (2)
Pr7—O25^xi^	2.465 (2)	Mo9—O19	2.064 (2)
Pr7—O27^i^	2.474 (2)	Mo9—O14	2.084 (2)
Pr7—O24^xi^	2.598 (2)	Mo9—O23^xii^	2.0935 (19)
Pr7—O18	2.675 (2)	Mo9—O24^xii^	2.105 (2)
Pr7—O17	2.810 (2)	Mo9—Mo8^xii^	2.6006 (6)
Pr7—O7	3.128 (2)	Mo9—Mo10	2.7168 (5)
Pr8—O27^ix^	2.309 (2)	Mo9—Mo10^xii^	2.7422 (4)
Pr8—O21^vii^	2.384 (2)	Mo10—O16^xii^	2.0182 (19)
Pr8—O9^ii^	2.389 (2)	Mo10—O21	2.062 (2)
Pr8—O3	2.442 (2)	Mo10—O23^xii^	2.066 (2)
Pr8—O7^ii^	2.509 (2)	Mo10—O24	2.086 (2)
Pr8—O20^vii^	2.700 (2)	Mo10—Mo6^xii^	2.7396 (6)
Pr8—O24^vii^	2.708 (2)	Mo10—Mo9^xii^	2.7422 (4)
Pr8—O4^ii^	3.235 (2)	Mo10—Mo8^xii^	2.7556 (5)
Pr8—O1	3.360 (2)	Mo10—Mo10^xii^	2.8525 (7)
Mo1—O1	1.939 (2)	Mo11—O5^vii^	2.024 (2)
Mo1—O4	2.004 (2)	Mo11—O5	2.024 (2)
Mo1—O3	2.009 (2)	Mo11—O9^xiii^	2.027 (2)
Mo1—O2	2.046 (2)	Mo11—O9^ii^	2.027 (2)
Mo1—O5	2.175 (2)	Mo11—O22	2.048 (2)
Mo1—Mo2	2.6435 (5)	Mo11—O22^vii^	2.048 (2)
			
O28^i^—Pr1—O22	127.49 (7)	O22^vii^—Pr5—O5^vii^	66.29 (6)
O28^i^—Pr1—O3	126.76 (7)	O8^ii^—Pr5—O9^ii^	63.29 (7)
O22—Pr1—O3	103.26 (7)	O27^vi^—Pr5—O9^ii^	143.01 (7)
O28^i^—Pr1—O2	109.90 (7)	O20^vii^—Pr5—O9^ii^	76.02 (7)
O22—Pr1—O2	73.14 (7)	O2^vii^—Pr5—O9^ii^	122.33 (7)
O3—Pr1—O2	66.81 (6)	O22^vii^—Pr5—O9^ii^	63.91 (6)
O28^i^—Pr1—O6^ii^	88.52 (7)	O5^vii^—Pr5—O9^ii^	61.94 (6)
O22—Pr1—O6^ii^	80.86 (7)	O8^ii^—Pr5—O4^vii^	62.59 (6)
O3—Pr1—O6^ii^	117.38 (6)	O27^vi^—Pr5—O4^vii^	84.51 (7)
O2—Pr1—O6^ii^	153.77 (6)	O20^vii^—Pr5—O4^vii^	171.27 (6)
O28^i^—Pr1—O10	65.55 (7)	O2^vii^—Pr5—O4^vii^	62.77 (6)
O22—Pr1—O10	138.50 (7)	O22^vii^—Pr5—O4^vii^	118.99 (6)
O3—Pr1—O10	65.76 (6)	O5^vii^—Pr5—O4^vii^	60.36 (6)
O2—Pr1—O10	65.73 (6)	O9^ii^—Pr5—O4^vii^	110.19 (6)
O6^ii^—Pr1—O10	140.47 (6)	O8^ii^—Pr5—O17^vii^	177.60 (6)
O28^i^—Pr1—O4^ii^	91.40 (7)	O27^vi^—Pr5—O17^vii^	82.68 (7)
O22—Pr1—O4^ii^	126.11 (7)	O20^vii^—Pr5—O17^vii^	60.44 (6)
O3—Pr1—O4^ii^	66.53 (7)	O2^vii^—Pr5—O17^vii^	60.08 (6)
O2—Pr1—O4^ii^	132.58 (6)	O22^vii^—Pr5—O17^vii^	56.68 (6)
O6^ii^—Pr1—O4^ii^	62.38 (6)	O5^vii^—Pr5—O17^vii^	110.88 (6)
O10—Pr1—O4^ii^	87.77 (6)	O9^ii^—Pr5—O17^vii^	114.88 (6)
O28^i^—Pr1—O9^ii^	147.24 (7)	O4^vii^—Pr5—O17^vii^	119.80 (5)
O22—Pr1—O9^ii^	65.77 (6)	O8^ii^—Pr5—O7^ii^	60.05 (6)
O3—Pr1—O9^ii^	63.39 (6)	O27^vi^—Pr5—O7^ii^	90.83 (7)
O2—Pr1—O9^ii^	102.62 (6)	O20^vii^—Pr5—O7^ii^	57.35 (6)
O6^ii^—Pr1—O9^ii^	62.42 (6)	O2^vii^—Pr5—O7^ii^	173.67 (6)
O10—Pr1—O9^ii^	127.98 (6)	O22^vii^—Pr5—O7^ii^	102.19 (6)
O4^ii^—Pr1—O9^ii^	62.71 (6)	O5^vii^—Pr5—O7^ii^	109.12 (6)
O28^i^—Pr1—O17	75.61 (7)	O9^ii^—Pr5—O7^ii^	52.31 (6)
O22—Pr1—O17	60.13 (6)	O4^vii^—Pr5—O7^ii^	121.07 (5)
O3—Pr1—O17	127.73 (6)	O17^vii^—Pr5—O7^ii^	117.69 (5)
O2—Pr1—O17	60.97 (6)	Mo11—Pr5—O7^ii^	89.33 (4)
O6^ii^—Pr1—O17	108.61 (6)	O25^ii^—Pr6—O11^iv^	123.47 (7)
O10—Pr1—O17	93.86 (6)	O25^ii^—Pr6—O16^viii^	139.50 (8)
O4^ii^—Pr1—O17	164.77 (6)	O11^iv^—Pr6—O16^viii^	96.98 (7)
O9^ii^—Pr1—O17	125.88 (6)	O25^ii^—Pr6—O12^iv^	74.38 (7)
O28^i^—Pr1—O19	74.16 (6)	O11^iv^—Pr6—O12^iv^	70.86 (7)
O22—Pr1—O19	58.45 (6)	O16^viii^—Pr6—O12^iv^	123.29 (7)
O3—Pr1—O19	158.71 (6)	O25^ii^—Pr6—O18^viii^	80.34 (7)
O2—Pr1—O19	112.85 (6)	O11^iv^—Pr6—O18^viii^	134.81 (7)
O6^ii^—Pr1—O19	53.05 (6)	O16^viii^—Pr6—O18^viii^	68.25 (7)
O10—Pr1—O19	134.76 (6)	O12^iv^—Pr6—O18^viii^	82.16 (7)
O4^ii^—Pr1—O19	113.61 (6)	O25^ii^—Pr6—O14^viii^	76.00 (7)
O9^ii^—Pr1—O19	97.08 (6)	O11^iv^—Pr6—O14^viii^	153.15 (6)
O17—Pr1—O19	55.63 (5)	O16^viii^—Pr6—O14^viii^	66.77 (6)
O28^i^—Pr1—Mo2^ii^	108.58 (5)	O12^iv^—Pr6—O14^viii^	135.75 (6)
O22—Pr1—Mo2^ii^	91.89 (5)	O18^viii^—Pr6—O14^viii^	60.94 (6)
O3—Pr1—Mo2^ii^	81.64 (5)	O25^ii^—Pr6—O27^ix^	100.30 (7)
O2—Pr1—Mo2^ii^	139.98 (5)	O11^iv^—Pr6—O27^ix^	71.10 (7)
O6^ii^—Pr1—Mo2^ii^	35.90 (5)	O16^viii^—Pr6—O27^ix^	94.09 (6)
O10—Pr1—Mo2^ii^	123.34 (5)	O12^iv^—Pr6—O27^ix^	128.86 (6)
O4^ii^—Pr1—Mo2^ii^	35.80 (4)	O18^viii^—Pr6—O27^ix^	148.32 (6)
O9^ii^—Pr1—Mo2^ii^	38.77 (4)	O14^viii^—Pr6—O27^ix^	88.24 (6)
O17—Pr1—Mo2^ii^	141.42 (4)	O25^ii^—Pr6—O13	127.69 (7)
O19—Pr1—Mo2^ii^	87.83 (4)	O11^iv^—Pr6—O13	65.22 (6)
O28^i^—Pr1—Mo11	157.97 (5)	O16^viii^—Pr6—O13	64.67 (6)
O22—Pr1—Mo11	33.56 (5)	O12^iv^—Pr6—O13	59.98 (6)
O3—Pr1—Mo11	75.23 (5)	O18^viii^—Pr6—O13	69.99 (6)
O2—Pr1—Mo11	78.39 (4)	O14^viii^—Pr6—O13	120.17 (6)
O6^ii^—Pr1—Mo11	78.06 (5)	O27^ix^—Pr6—O13	127.38 (6)
O10—Pr1—Mo11	134.58 (4)	O25^ii^—Pr6—O7^iv^	70.87 (7)
O4^ii^—Pr1—Mo11	97.58 (4)	O11^iv^—Pr6—O7^iv^	52.60 (6)
O9^ii^—Pr1—Mo11	34.87 (4)	O16^viii^—Pr6—O7^iv^	149.53 (6)
O17—Pr1—Mo11	92.05 (4)	O12^iv^—Pr6—O7^iv^	51.17 (6)
O19—Pr1—Mo11	83.81 (4)	O18^viii^—Pr6—O7^iv^	129.72 (6)
Mo2^ii^—Pr1—Mo11	69.936 (13)	O14^viii^—Pr6—O7^iv^	141.24 (5)
O28^i^—Pr1—Mo1	129.95 (5)	O27^ix^—Pr6—O7^iv^	78.60 (6)
O22—Pr1—Mo1	82.99 (5)	O13—Pr6—O7^iv^	96.22 (5)
O3—Pr1—Mo1	33.39 (5)	Mo6^viii^—Pr6—O7^iv^	170.08 (4)
O2—Pr1—Mo1	34.32 (5)	Mo3^iv^—Pr6—O7^iv^	34.29 (3)
O6^ii^—Pr1—Mo1	139.62 (5)	O25^ii^—Pr6—O24^vii^	114.48 (6)
O10—Pr1—Mo1	66.38 (4)	O11^iv^—Pr6—O24^vii^	101.71 (6)
O4^ii^—Pr1—Mo1	99.91 (5)	O16^viii^—Pr6—O24^vii^	51.31 (6)
O9^ii^—Pr1—Mo1	77.20 (4)	O12^iv^—Pr6—O24^vii^	171.00 (6)
O17—Pr1—Mo1	94.60 (4)	O18^viii^—Pr6—O24^vii^	100.54 (5)
O19—Pr1—Mo1	138.91 (4)	O14^viii^—Pr6—O24^vii^	51.45 (5)
Mo2^ii^—Pr1—Mo1	108.660 (15)	O27^ix^—Pr6—O24^vii^	50.00 (5)
Mo11—Pr1—Mo1	68.271 (13)	O13—Pr6—O24^vii^	112.68 (5)
O28^i^—Pr1—Mo7	92.49 (5)	Mo6^viii^—Pr6—O24^vii^	59.82 (3)
O22—Pr1—Mo7	35.10 (5)	Mo3^iv^—Pr6—O24^vii^	137.95 (3)
O3—Pr1—Mo7	134.84 (5)	O7^iv^—Pr6—O24^vii^	128.58 (5)
O2—Pr1—Mo7	80.32 (5)	O12—Pr7—O26^x^	121.27 (7)
O6^ii^—Pr1—Mo7	80.38 (5)	O12—Pr7—O15	131.23 (7)
O10—Pr1—Mo7	127.47 (5)	O26^x^—Pr7—O15	68.32 (7)
O4^ii^—Pr1—Mo7	142.43 (4)	O12—Pr7—O25^xi^	73.01 (7)
O9^ii^—Pr1—Mo7	96.80 (4)	O26^x^—Pr7—O25^xi^	65.97 (7)
O17—Pr1—Mo7	33.62 (4)	O15—Pr7—O25^xi^	134.12 (7)
O19—Pr1—Mo7	33.58 (4)	O12—Pr7—O27^i^	116.68 (7)
Mo2^ii^—Pr1—Mo7	108.607 (15)	O26^x^—Pr7—O27^i^	120.92 (7)
Mo11—Pr1—Mo7	68.262 (15)	O15—Pr7—O27^i^	82.68 (7)
Mo1—Pr1—Mo7	105.888 (13)	O25^xi^—Pr7—O27^i^	124.88 (7)
O28^i^—Pr1—O5	160.79 (6)	O12—Pr7—O24^xi^	127.76 (7)
O22—Pr1—O5	52.26 (6)	O26^x^—Pr7—O24^xi^	70.12 (7)
O3—Pr1—O5	51.44 (6)	O15—Pr7—O24^xi^	100.91 (6)
O2—Pr1—O5	50.90 (6)	O25^xi^—Pr7—O24^xi^	67.07 (6)
O6^ii^—Pr1—O5	109.36 (6)	O27^i^—Pr7—O24^xi^	66.43 (6)
O10—Pr1—O5	101.84 (5)	O12—Pr7—O18	71.75 (7)
O4^ii^—Pr1—O5	102.93 (6)	O26^x^—Pr7—O18	67.74 (7)
O9^ii^—Pr1—O5	51.91 (5)	O15—Pr7—O18	69.78 (6)
O17—Pr1—O5	91.56 (6)	O25^xi^—Pr7—O18	89.33 (7)
O19—Pr1—O5	110.57 (5)	O27^i^—Pr7—O18	145.73 (6)
Mo2^ii^—Pr1—O5	90.39 (4)	O24^xi^—Pr7—O18	137.26 (6)
Mo11—Pr1—O5	32.94 (3)	O12—Pr7—O17	73.63 (7)
Mo1—Pr1—O5	35.48 (3)	O26^x^—Pr7—O17	119.09 (6)
Mo7—Pr1—O5	83.96 (4)	O15—Pr7—O17	62.76 (6)
O28^i^—Pr1—Mo5	96.06 (5)	O25^xi^—Pr7—O17	141.92 (6)
O22—Pr1—Mo5	127.01 (5)	O27^i^—Pr7—O17	86.69 (7)
O3—Pr1—Mo5	32.42 (4)	O24^xi^—Pr7—O17	150.79 (6)
O2—Pr1—Mo5	63.50 (4)	O18—Pr7—O17	62.98 (6)
O6^ii^—Pr1—Mo5	134.99 (5)	O12—Pr7—O7	59.55 (6)
O10—Pr1—Mo5	33.47 (5)	O26^x^—Pr7—O7	139.28 (6)
O4^ii^—Pr1—Mo5	72.74 (4)	O15—Pr7—O7	146.36 (6)
O9^ii^—Pr1—Mo5	94.82 (5)	O25^xi^—Pr7—O7	77.56 (7)
O17—Pr1—Mo5	115.92 (4)	O27^i^—Pr7—O7	66.56 (7)
O19—Pr1—Mo5	168.08 (4)	O24^xi^—Pr7—O7	79.67 (6)
Mo2^ii^—Pr1—Mo5	101.978 (15)	O18—Pr7—O7	131.30 (6)
Mo11—Pr1—Mo5	105.800 (16)	O17—Pr7—O7	100.65 (6)
Mo1—Pr1—Mo5	44.065 (7)	O12—Pr7—O2	54.92 (6)
Mo7—Pr1—Mo5	143.620 (9)	O26^x^—Pr7—O2	168.96 (6)
O5—Pr1—Mo5	76.49 (3)	O15—Pr7—O2	105.81 (6)
O13^ii^—Pr2—O26	67.88 (7)	O25^xi^—Pr7—O2	118.50 (6)
O13^ii^—Pr2—O28^i^	125.37 (8)	O27^i^—Pr7—O2	65.95 (6)
O26—Pr2—O28^i^	142.27 (7)	O24^xi^—Pr7—O2	120.76 (6)
O13^ii^—Pr2—O16^iii^	71.49 (7)	O18—Pr7—O2	101.66 (6)
O26—Pr2—O16^iii^	99.88 (7)	O17—Pr7—O2	50.72 (5)
O28^i^—Pr2—O16^iii^	117.70 (7)	O7—Pr7—O2	50.07 (5)
O13^ii^—Pr2—O15^iii^	109.90 (7)	O27^ix^—Pr8—O21^vii^	123.74 (7)
O26—Pr2—O15^iii^	66.68 (7)	O27^ix^—Pr8—O9^ii^	137.61 (7)
O28^i^—Pr2—O15^iii^	123.51 (7)	O21^vii^—Pr8—O9^ii^	98.57 (7)
O16^iii^—Pr2—O15^iii^	67.30 (7)	O27^ix^—Pr8—O3	97.20 (7)
O13^ii^—Pr2—O23^iii^	135.72 (7)	O21^vii^—Pr8—O3	104.08 (7)
O26—Pr2—O23^iii^	131.32 (7)	O9^ii^—Pr8—O3	72.33 (7)
O28^i^—Pr2—O23^iii^	67.70 (7)	O27^ix^—Pr8—O7^ii^	80.67 (7)
O16^iii^—Pr2—O23^iii^	66.23 (6)	O21^vii^—Pr8—O7^ii^	131.05 (7)
O15^iii^—Pr2—O23^iii^	64.98 (6)	O9^ii^—Pr8—O7^ii^	68.21 (7)
O13^ii^—Pr2—O14	129.30 (7)	O3—Pr8—O7^ii^	114.77 (7)
O26—Pr2—O14	65.80 (6)	O27^ix^—Pr8—O20^vii^	115.06 (7)
O28^i^—Pr2—O14	83.74 (7)	O21^vii^—Pr8—O20^vii^	64.91 (6)
O16^iii^—Pr2—O14	135.75 (7)	O9^ii^—Pr8—O20^vii^	78.45 (7)
O15^iii^—Pr2—O14	68.66 (7)	O3—Pr8—O20^vii^	146.87 (6)
O23^iii^—Pr2—O14	91.45 (6)	O7^ii^—Pr8—O20^vii^	66.27 (7)
O13^ii^—Pr2—O19	79.01 (7)	O27^ix^—Pr8—O24^vii^	66.76 (7)
O26—Pr2—O19	63.96 (7)	O21^vii^—Pr8—O24^vii^	68.35 (6)
O28^i^—Pr2—O19	83.00 (7)	O9^ii^—Pr8—O24^vii^	137.75 (7)
O16^iii^—Pr2—O19	150.17 (6)	O3—Pr8—O24^vii^	148.66 (6)
O15^iii^—Pr2—O19	121.01 (6)	O7^ii^—Pr8—O24^vii^	89.96 (7)
O23^iii^—Pr2—O19	143.45 (6)	O20^vii^—Pr8—O24^vii^	59.47 (6)
O14—Pr2—O19	63.07 (6)	O27^ix^—Pr8—O4^ii^	79.08 (7)
O13^ii^—Pr2—O1^ii^	64.64 (7)	O21^vii^—Pr8—O4^ii^	154.64 (6)
O26—Pr2—O1^ii^	132.38 (6)	O9^ii^—Pr8—O4^ii^	60.23 (6)
O28^i^—Pr2—O1^ii^	70.05 (7)	O3—Pr8—O4^ii^	58.26 (6)
O16^iii^—Pr2—O1^ii^	68.49 (7)	O7^ii^—Pr8—O4^ii^	57.42 (6)
O15^iii^—Pr2—O1^ii^	134.45 (6)	O20^vii^—Pr8—O4^ii^	118.58 (6)
O23^iii^—Pr2—O1^ii^	87.66 (6)	O24^vii^—Pr8—O4^ii^	136.45 (6)
O14—Pr2—O1^ii^	152.00 (6)	O27^ix^—Pr8—O1	102.21 (7)
O19—Pr2—O1^ii^	102.86 (6)	O21^vii^—Pr8—O1	53.99 (6)
O13^ii^—Pr2—O6^ii^	57.69 (7)	O9^ii^—Pr8—O1	105.11 (6)
O26—Pr2—O6^ii^	98.05 (6)	O3—Pr8—O1	56.95 (6)
O28^i^—Pr2—O6^ii^	71.17 (6)	O7^ii^—Pr8—O1	171.34 (6)
O16^iii^—Pr2—O6^ii^	113.26 (6)	O20^vii^—Pr8—O1	118.74 (6)
O15^iii^—Pr2—O6^ii^	164.08 (5)	O24^vii^—Pr8—O1	98.69 (6)
O23^iii^—Pr2—O6^ii^	130.57 (6)	O4^ii^—Pr8—O1	114.81 (5)
O14—Pr2—O6^ii^	110.22 (6)	O27^ix^—Pr8—O5	146.50 (6)
O19—Pr2—O6^ii^	50.28 (6)	O21^vii^—Pr8—O5	59.73 (6)
O1^ii^—Pr2—O6^ii^	52.69 (5)	O9^ii^—Pr8—O5	56.10 (6)
Mo9—Pr2—O6^ii^	87.51 (4)	O3—Pr8—O5	53.80 (6)
O5^ii^—Pr3—O28^i^	131.27 (7)	O7^ii^—Pr8—O5	124.13 (6)
O5^ii^—Pr3—O6^iv^	99.08 (7)	O20^vii^—Pr8—O5	96.76 (5)
O28^i^—Pr3—O6^iv^	119.26 (7)	O24^vii^—Pr8—O5	128.08 (5)
O5^ii^—Pr3—O19^i^	119.46 (7)	O4^ii^—Pr8—O5	95.30 (5)
O28^i^—Pr3—O19^i^	104.17 (7)	O1—Pr8—O5	50.03 (5)
O6^iv^—Pr3—O19^i^	65.16 (7)	Mo2^ii^—Pr8—O5	93.39 (4)
O5^ii^—Pr3—O21^i^	77.54 (7)	O27^ix^—Pr8—O11^iv^	57.68 (6)
O28^i^—Pr3—O21^i^	103.22 (7)	O21^vii^—Pr8—O11^iv^	96.93 (6)
O6^iv^—Pr3—O21^i^	121.28 (6)	O9^ii^—Pr8—O11^iv^	125.09 (6)
O19^i^—Pr3—O21^i^	66.59 (6)	O3—Pr8—O11^iv^	52.83 (6)
O5^ii^—Pr3—O10	118.03 (7)	O7^ii^—Pr8—O11^iv^	129.91 (6)
O28^i^—Pr3—O10	61.25 (6)	O20^vii^—Pr8—O11^iv^	153.60 (5)
O6^iv^—Pr3—O10	66.35 (6)	O24^vii^—Pr8—O11^iv^	96.83 (5)
O19^i^—Pr3—O10	107.91 (6)	O4^ii^—Pr8—O11^iv^	86.23 (5)
O21^i^—Pr3—O10	162.66 (6)	O1—Pr8—O11^iv^	48.79 (5)
O5^ii^—Pr3—O1^ii^	66.50 (6)	Mo2^ii^—Pr8—O11^iv^	121.40 (3)
O28^i^—Pr3—O1^ii^	71.44 (7)	O5—Pr8—O11^iv^	89.18 (5)
O6^iv^—Pr3—O1^ii^	164.96 (7)	O1—Mo1—O4	90.21 (9)
O19^i^—Pr3—O1^ii^	124.77 (6)	O1—Mo1—O3	93.74 (9)
O21^i^—Pr3—O1^ii^	61.64 (6)	O4—Mo1—O3	169.69 (8)
O10—Pr3—O1^ii^	115.58 (6)	O1—Mo1—O2	171.74 (8)
O5^ii^—Pr3—O8^iv^	64.79 (7)	O4—Mo1—O2	89.51 (9)
O28^i^—Pr3—O8^iv^	106.97 (7)	O3—Mo1—O2	85.22 (8)
O6^iv^—Pr3—O8^iv^	61.22 (6)	O1—Mo1—O5	88.83 (8)
O19^i^—Pr3—O8^iv^	125.94 (6)	O4—Mo1—O5	85.93 (8)
O21^i^—Pr3—O8^iv^	141.59 (6)	O3—Mo1—O5	84.64 (8)
O10—Pr3—O8^iv^	55.39 (6)	O2—Mo1—O5	82.92 (8)
O1^ii^—Pr3—O8^iv^	106.69 (6)	O7—Mo2—O8	96.31 (9)
O5^ii^—Pr3—O22^i^	61.50 (6)	O7—Mo2—O4	90.68 (9)
O28^i^—Pr3—O22^i^	157.42 (6)	O8—Mo2—O4	168.06 (9)
O6^iv^—Pr3—O22^i^	68.85 (6)	O7—Mo2—O6	169.07 (8)
O19^i^—Pr3—O22^i^	58.31 (6)	O8—Mo2—O6	85.49 (9)
O21^i^—Pr3—O22^i^	58.19 (6)	O4—Mo2—O6	85.86 (9)
O10—Pr3—O22^i^	134.41 (5)	O7—Mo2—O9	83.26 (8)
O1^ii^—Pr3—O22^i^	105.53 (6)	O8—Mo2—O9	82.55 (8)
O8^iv^—Pr3—O22^i^	95.39 (6)	O4—Mo2—O9	88.70 (8)
O5^ii^—Pr3—O23^iii^	131.48 (6)	O6—Mo2—O9	86.30 (8)
O28^i^—Pr3—O23^iii^	57.46 (6)	O12—Mo3—O2	92.52 (8)
O6^iv^—Pr3—O23^iii^	116.05 (6)	O12—Mo3—O7	85.81 (8)
O19^i^—Pr3—O23^iii^	57.29 (6)	O2—Mo3—O7	88.20 (8)
O21^i^—Pr3—O23^iii^	56.20 (6)	O12—Mo3—O10	91.26 (8)
O10—Pr3—O23^iii^	106.66 (5)	O2—Mo3—O10	84.33 (8)
O1^ii^—Pr3—O23^iii^	78.28 (6)	O7—Mo3—O10	171.84 (8)
O8^iv^—Pr3—O23^iii^	161.96 (5)	O12—Mo3—O11	85.92 (8)
O22^i^—Pr3—O23^iii^	99.97 (5)	O2—Mo3—O11	174.12 (8)
O5^ii^—Pr3—O4^ii^	58.38 (6)	O7—Mo3—O11	86.04 (8)
O28^i^—Pr3—O4^ii^	77.51 (6)	O10—Mo3—O11	101.36 (8)
O6^iv^—Pr3—O4^ii^	115.52 (6)	O13—Mo4—O1	89.13 (8)
O19^i^—Pr3—O4^ii^	177.68 (6)	O13—Mo4—O11^iv^	87.65 (9)
O21^i^—Pr3—O4^ii^	111.58 (6)	O1—Mo4—O11^iv^	85.76 (8)
O10—Pr3—O4^ii^	74.26 (5)	O13—Mo4—O6	90.85 (9)
O1^ii^—Pr3—O4^ii^	54.08 (6)	O1—Mo4—O6	84.84 (8)
O8^iv^—Pr3—O4^ii^	54.48 (5)	O11^iv^—Mo4—O6	170.50 (8)
O22^i^—Pr3—O4^ii^	119.64 (5)	O13—Mo4—O10^iv^	87.45 (8)
O23^iii^—Pr3—O4^ii^	123.16 (5)	O1—Mo4—O10^iv^	171.74 (8)
O18—Pr4—O26^i^	123.01 (7)	O11^iv^—Mo4—O10^iv^	101.60 (7)
O18—Pr4—O13^iv^	132.96 (8)	O6—Mo4—O10^iv^	87.69 (8)
O26^i^—Pr4—O13^iv^	66.63 (7)	O8^iv^—Mo5—O11^iv^	90.91 (9)
O18—Pr4—O12	75.77 (7)	O8^iv^—Mo5—O3	89.91 (8)
O26^i^—Pr4—O12	126.69 (7)	O11^iv^—Mo5—O3	86.69 (8)
O13^iv^—Pr4—O12	66.55 (7)	O8^iv^—Mo5—O10	80.79 (8)
O18—Pr4—O28^i^	116.60 (7)	O11^iv^—Mo5—O10	168.31 (8)
O26^i^—Pr4—O28^i^	111.05 (7)	O3—Mo5—O10	85.09 (8)
O13^iv^—Pr4—O28^i^	96.05 (7)	O14—Mo6—O17	94.99 (9)
O12—Pr4—O28^i^	97.80 (7)	O14—Mo6—O15	172.30 (8)
O18—Pr4—O25^iii^	75.56 (7)	O17—Mo6—O15	86.89 (9)
O26^i^—Pr4—O25^iii^	61.52 (6)	O14—Mo6—O16	91.07 (9)
O13^iv^—Pr4—O25^iii^	127.77 (6)	O17—Mo6—O16	169.53 (8)
O12—Pr4—O25^iii^	148.06 (7)	O15—Mo6—O16	86.01 (8)
O28^i^—Pr4—O25^iii^	107.41 (7)	O14—Mo6—O18	82.97 (8)
O18—Pr4—O18^v^	73.76 (7)	O17—Mo6—O18	87.88 (8)
O26^i^—Pr4—O18^v^	64.37 (6)	O15—Mo6—O18	89.65 (8)
O13^iv^—Pr4—O18^v^	71.75 (7)	O16—Mo6—O18	84.39 (8)
O12—Pr4—O18^v^	77.96 (6)	O17—Mo7—O20	91.42 (9)
O28^i^—Pr4—O18^v^	167.80 (6)	O17—Mo7—O19	94.23 (9)
O25^iii^—Pr4—O18^v^	80.80 (6)	O20—Mo7—O19	169.39 (8)
O18—Pr4—O23^iii^	123.28 (6)	O17—Mo7—O21	169.19 (8)
O26^i^—Pr4—O23^iii^	61.01 (6)	O20—Mo7—O21	86.37 (8)
O13^iv^—Pr4—O23^iii^	102.05 (7)	O19—Mo7—O21	86.41 (8)
O12—Pr4—O23^iii^	154.05 (6)	O17—Mo7—O22	83.83 (8)
O28^i^—Pr4—O23^iii^	59.01 (6)	O20—Mo7—O22	85.49 (8)
O25^iii^—Pr4—O23^iii^	57.50 (6)	O19—Mo7—O22	86.20 (8)
O18^v^—Pr4—O23^iii^	122.07 (6)	O21—Mo7—O22	85.45 (8)
O18—Pr4—O14	55.49 (6)	O25—Mo8—O15	92.08 (8)
O26^i^—Pr4—O14	120.34 (6)	O25—Mo8—O20	91.90 (8)
O13^iv^—Pr4—O14	166.13 (7)	O15—Mo8—O20	92.72 (8)
O12—Pr4—O14	111.11 (6)	O25—Mo8—O24	85.85 (8)
O28^i^—Pr4—O14	70.44 (6)	O15—Mo8—O24	172.66 (8)
O25^iii^—Pr4—O14	61.83 (6)	O20—Mo8—O24	80.33 (8)
O18^v^—Pr4—O14	121.76 (6)	O25—Mo8—O23	85.50 (8)
O23^iii^—Pr4—O14	74.09 (5)	O15—Mo8—O23	83.56 (8)
O18—Pr4—O17	56.09 (6)	O20—Mo8—O23	175.37 (8)
O26^i^—Pr4—O17	171.82 (6)	O24—Mo8—O23	103.26 (8)
O13^iv^—Pr4—O17	120.52 (6)	O26—Mo9—O19	85.50 (8)
O12—Pr4—O17	61.48 (6)	O26—Mo9—O14	86.05 (8)
O28^i^—Pr4—O17	65.61 (6)	O19—Mo9—O14	86.13 (8)
O25^iii^—Pr4—O17	111.66 (6)	O26—Mo9—O23^xii^	88.03 (8)
O18^v^—Pr4—O17	120.47 (6)	O19—Mo9—O23^xii^	85.27 (8)
O23^iii^—Pr4—O17	111.96 (5)	O14—Mo9—O23^xii^	169.91 (8)
O14—Pr4—O17	51.77 (5)	O26—Mo9—O24^xii^	89.13 (8)
O18—Pr4—O10	123.77 (6)	O19—Mo9—O24^xii^	170.52 (8)
O26^i^—Pr4—O10	109.97 (6)	O14—Mo9—O24^xii^	85.71 (8)
O13^iv^—Pr4—O10	54.02 (6)	O23^xii^—Mo9—O24^xii^	102.38 (8)
O12—Pr4—O10	57.27 (6)	O16^xii^—Mo10—O21	90.25 (8)
O28^i^—Pr4—O10	50.75 (6)	O16^xii^—Mo10—O23^xii^	85.71 (8)
O25^iii^—Pr4—O10	154.38 (5)	O21—Mo10—O23^xii^	84.30 (8)
O18^v^—Pr4—O10	118.81 (5)	O16^xii^—Mo10—O24	85.99 (8)
O23^iii^—Pr4—O10	96.94 (5)	O21—Mo10—O24	87.67 (8)
O14—Pr4—O10	112.67 (5)	O23^xii^—Mo10—O24	168.42 (8)
O17—Pr4—O10	74.12 (5)	O5^vii^—Mo11—O5	180.000 (1)
O8^ii^—Pr5—O27^vi^	97.98 (8)	O5^vii^—Mo11—O9^xiii^	91.74 (8)
O8^ii^—Pr5—O20^vii^	117.25 (7)	O5—Mo11—O9^xiii^	88.26 (8)
O27^vi^—Pr5—O20^vii^	86.91 (8)	O5^vii^—Mo11—O9^ii^	88.26 (8)
O8^ii^—Pr5—O2^vii^	122.07 (7)	O5—Mo11—O9^ii^	91.74 (8)
O27^vi^—Pr5—O2^vii^	94.65 (7)	O9^xiii^—Mo11—O9^ii^	180.00 (6)
O20^vii^—Pr5—O2^vii^	119.72 (7)	O5^vii^—Mo11—O22	91.39 (8)
O8^ii^—Pr5—O22^vii^	122.38 (7)	O5—Mo11—O22	88.61 (8)
O27^vi^—Pr5—O22^vii^	138.92 (7)	O9^xiii^—Mo11—O22	89.70 (9)
O20^vii^—Pr5—O22^vii^	68.98 (7)	O9^ii^—Mo11—O22	90.30 (9)
O2^vii^—Pr5—O22^vii^	71.56 (7)	O5^vii^—Mo11—O22^vii^	88.61 (8)
O8^ii^—Pr5—O5^vii^	69.86 (7)	O5—Mo11—O22^vii^	91.39 (8)
O27^vi^—Pr5—O5^vii^	144.73 (7)	O9^xiii^—Mo11—O22^vii^	90.30 (9)
O20^vii^—Pr5—O5^vii^	128.29 (7)	O9^ii^—Mo11—O22^vii^	89.70 (9)
O2^vii^—Pr5—O5^vii^	67.77 (7)	O22—Mo11—O22^vii^	180.0

Symmetry codes: (i) *x*, −*y*+1/2, *z*−1/2; (ii) −*x*+1, *y*+1/2, −*z*+1/2; (iii) −*x*, *y*+1/2, −*z*+1/2; (iv) −*x*+1, −*y*, −*z*; (v) −*x*, −*y*, −*z*; (vi) −*x*+1, *y*−1/2, −*z*+3/2; (vii) −*x*+1, −*y*, −*z*+1; (viii) *x*+1, *y*, *z*; (ix) −*x*+1, −*y*+1, −*z*+1; (x) −*x*, *y*−1/2, −*z*+1/2; (xi) *x*, −*y*−1/2, *z*−1/2; (xii) −*x*, −*y*, −*z*+1; (xiii) *x*, −*y*−1/2, *z*+1/2.
